# Preoperative Parameters Associated with Vitrectomy Outcomes in Myopic Traction Maculopathy Without a Full-Thickness Macular Hole

**DOI:** 10.3390/life16020356

**Published:** 2026-02-19

**Authors:** Su Kyung Lee, Suji Yeo, Yoo-Ri Chung, Hae Rang Kim, Ji Hun Song

**Affiliations:** Department of Ophthalmology, Ajou University School of Medicine, Suwon 16499, Gyeonggi-do, Republic of Korea; sukyung126@gmail.com (S.K.L.); ysj0326@aumc.ac.kr (S.Y.); khr1412@hanmail.net (H.R.K.)

**Keywords:** ellipsoid zone disruption, foveoschisis, foveal detachment, internal limiting membrane peeling, myopic traction maculopathy, pathologic myopia, vitrectomy

## Abstract

Pathologic myopia has become a major global cause of blindness, making timely surgical management for myopic traction maculopathy (MTM) increasingly important. This study aimed to identify prognostic factors associated with functional and anatomical outcomes following surgery for MTM and to determine the optimal timing for intervention. This retrospective study included 33 eyes from 28 patients with MTM without full-thickness macular hole who underwent pars plana vitrectomy with internal limiting membrane peeling and gas tamponade. Better preoperative best-corrected visual acuity (BCVA) and lower foveal height were associated with better postoperative BCVA, whereas longer axial length, higher MTM, and higher Atrophy–Traction–Neovascularization (ATN) classification grade were correlated with thinner postoperative central foveal thickness. Foveal detachment (FD), ellipsoid zone (EZ) disruption, and advanced MTM grade were associated with poorer functional and anatomical outcomes. Postoperative visual outcomes should be interpreted with caution, as they may have been influenced by lens-related factors, including combined cataract surgery, post-vitrectomy cataract progression, and posterior capsule opacity. Nonetheless, consistent anatomical improvement was observed, supporting early surgical consideration in eyes with MTM showing progressive macular traction or EZ disruption, even in the absence of FD. These findings highlight the importance of serial OCT monitoring and individualized surgical timing based on preoperative assessments.

## 1. Introduction

Myopia has become a major global health concern, particularly in East Asia, and is projected to affect approximately 50% of the world’s population (with 10% developing high myopia) by 2050 [1,2]. Although the precise cutoff for “high myopia” remains debated, it is generally defined as a spherical equivalent (SE) of ≤−6.0 diopters (D) [3]. Clinically, axial length (AXL) thresholds such as >26.0 or 26.5 mm are also commonly used to characterize highly myopic eyes, although no universally accepted cutoff exists. Pathologic myopia (PM), characterized by excessive axial elongation leading to structural changes in the posterior segment [3,4,5], is a major cause of irreversible visual loss, accounting for 5.8–7.8% of blindness in Europe [6] and 12–27% in Asia. Myopic maculopathy refers to macular alterations induced by PM, including tessellated fundus, diffuse or patchy chorioretinal atrophy, and macular atrophy [4,7].

Myopic traction maculopathy (MTM), first described by Panozzo and Mercanti in 2004 [8], encompasses a spectrum of tractional changes affecting the macula in highly myopic eyes. MTM includes foveoschisis, retinal or foveal detachment (RD/FD), lamellar macular hole (LMH), and full-thickness macular hole (FTMH) with or without RD [4,9]. MTM occurs in up to 30% of eyes with PM [9]. Although early-stage MTM may remain stable or even resolve spontaneously [10], approximately 35–70% of eyes with foveoschisis progress to FD [8,10,11,12]. Once FD develops, MTM can rapidly evolve into FTMH or macular hole-related retinal detachment (MHRD), often resulting in irreversible visual loss [13]. Therefore, timely surgical intervention is essential to prevent permanent visual decline in progressive MTM. Various surgical techniques have been proposed for MTM, including posterior scleral reinforcement to limit axial elongation [14], macular buckling to counteract posterior staphyloma [15,16,17,18,19,20], and pars plana vitrectomy (PPV) to relieve vitreoretinal traction [21]. PPV was first introduced by Gonvers and Machemer in 1982 for MHRD [21] and has since evolved to include internal limiting membrane (ILM) peeling (either complete or fovea-sparing) combined with gas or silicone oil tamponade to enhance anatomic reattachment and visual outcomes [4,9]. Although the safety and efficacy of fovea-sparing ILM peeling remain controversial, PPV has nonetheless become the most commonly performed surgical procedure for MTM in recent years [22,23,24,25].

Determining the optimal timing of surgery to achieve favorable long-term outcomes in MTM remains highly debated. Anatomical worsening on optical coherence tomography (OCT), progressive visual decline, and the presence of vitreomacular traction (VMT), epiretinal membrane (ERM), foveoschisis, or FD are commonly considered surgical indications. However, the relative prognostic significance of these factors and the threshold levels at which surgery should be considered remain uncertain. Consequently, decisions regarding the timing of intervention are often individualized and based largely on clinical judgment rather than robust evidence. Previous studies [12,13] have mainly focused on visual prognosis based on the presence or absence of specific structural features (e.g., FD, VMT, LMH) rather than overall MTM severity at baseline, and structural outcomes have been assessed dichotomously, focusing only on retinal reattachment or FTMH development, which reflects surgical success versus failure.

Therefore, this study aimed to investigate the associations between preoperative factors (including quantitative OCT findings) and postoperative anatomical and visual outcomes after PPV with complete or fovea-sparing ILM peeling and gas tamponade in MTM eyes without FTMH. By analyzing these prognostic factors, we further aimed to determine the most appropriate timing for surgical intervention and to provide insights that support individualized management strategies for MTM.

## 2. Materials and Methods

This retrospective observational study was approved by the Institutional Review Board of Ajou University School of Medicine (AJOUIRB-DB-2025-511) and adhered to the tenets of the Declaration of Helsinki. The medical records of 33 eyes from 28 patients diagnosed with MTM without FTMH were reviewed. All patients underwent 25-gauge PPV combined with either complete or fovea-sparing ILM peeling and gas tamponade, performed by a single retinal surgeon (J.H.S.) at Ajou University Hospital between January 2013 and January 2024.

The inclusion criteria were as follows: (1) presence of foveoschisis, FD, or LMH with or without tractional components confirmed on OCT; (2) high myopia defined as an AXL of ≥26.0 mm or refractive error calculated as SE of ≤−6.0 D (the latter applied only to eyes without prior refractive or cataract surgery); (3) absence of active choroidal neovascularization or complete macular atrophy; and (4) progressive visual impairment associated with foveoschisis or FD.

The exclusion criteria were as follows: (1) presence of FTMH with or without RD; (2) postoperative follow-up period < 12 months; (3) history of previous vitreoretinal surgery; and (4) coexisting retinal or choroidal diseases that could affect visual function, including diabetic retinopathy, retinal vascular occlusions, neovascular age-related macular degeneration, central serous chorioretinopathy, or inflammatory ocular disorders.

### 2.1. Ophthalmic Examinations

All patients underwent comprehensive ophthalmologic examinations before and after surgery, including best-corrected visual acuity (BCVA), refractive error, intraocular pressure (NT-510; Nidek, Gamagori, Japan), slit-lamp biomicroscopy, and dilated fundus examination. Refractive error was measured with an auto-refractometer (GR-3500KA; Grand Seiko, Fukuyama, Japan, or RK-F2; Canon, Tokyo, Japan), and SE was calculated as the spherical value plus one-half of cylindrical value. AXL was obtained using IOL Master 500 (Carl Zeiss Meditec, Jena, Germany). BCVA was converted to the logarithm of the minimum angle of resolution (logMAR) for analysis.

**Figure 1:**
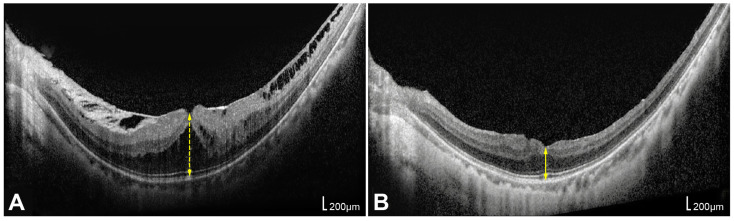
Representative optical coherence tomography (OCT) images of the same eye demonstrating the measurement of foveal height (FH) before surgery and central foveal thickness (CFT) after surgery. (**A**) FH was defined as the shortest vertical distance between the vitreoretinal interface and the outer border of the retinal pigment epithelium on preoperative OCT, reflecting traction-induced foveal deformation, including retinoschisis cavities and foveal detachment, as indicated by yellow dotted arrow lines. (**B**) CFT was defined as the minimum retinal thickness at the foveal center on postoperative OCT, representing the true retinal tissue thickness after resolution of schisis and reabsorption of subretinal fluid, as indicated by yellow arrow lines.

Spectral-domain OCT (Spectralis; Heidelberg Engineering, Heidelberg, Germany) and wide-field fundus photography (Optos; Optos plc, Dunfermline, UK) were performed. Foveal height (FH) was defined as the shortest vertical distance between the vitreoretinal interface and the outer border of the retinal pigment epithelium at the foveal center on preoperative OCT, reflecting traction-induced foveal deformation, including retinoschisis cavities and foveal detachment. Central foveal thickness (CFT) was measured as the minimum retinal thickness at the foveal center on postoperative OCT, representing the true retinal tissue thickness after resolution of schisis and reabsorption of subretinal fluid. Representative cases for measuring FH and CFT are shown in Figure 1.

### 2.2. Classification of Myopic Maculopathy

The ATN classification system, proposed by Ruiz-Medrano et al. [5], was used to grade myopic maculopathy based on the following three characteristics: atrophy (A), traction (T), and neovascularization (N). Grading criteria were as follows: A0, no myopic retinal lesions; A1, tessellated fundus only; A2, diffuse chorioretinal atrophy; A3, patchy chorioretinal atrophy; and A4, complete macular atrophy. T0, no macular schisis; T1, inner or outer foveoschisis; T2, combined inner and outer foveoschisis; T3, FD; T4, FTMH; and T5, macular hole with RD. N0, no myopic choroidal neovascularization; N1, macular lacquer cracks; N2a, active choroidal neovascularization; and N2s, scar or Fuchs’ spot. ATN total score was defined as the sum of individual A, T, and N grades; for example, an eye classified as A2T2N1 corresponded to an ATN total score of 5. All OCT and fundus images were independently evaluated by two retinal specialists (S.K.L. and S.Y.). In cases of disagreement, a third investigator (J.H.S.) determined the final grade.

### 2.3. Surgical Procedures

All surgical procedures were performed by a single experienced retinal specialist (J.H.S.) using standard 3-port 25-gauge PPV technique with the Constellation Vision System (Alcon, Fort Worth, TX, USA) under retrobulbar or general anesthesia. After core vitrectomy, triamcinolone acetonide (40 mg/mL) was injected to facilitate visualization and removal of the posterior hyaloid. Peripheral vitreous was subsequently removed to complete vitrectomy. ILM was stained using indocyanine green (0.25 mg/mL). If ERM was present, it was removed first; then ILM peeling was conducted using intraocular forceps over an area including the fovea and extending to the vascular arcade. A fovea-sparing technique was selectively applied in eyes with advanced MTM exhibiting extreme foveal thinning or lamellar hole. Complete ILM peeling was performed in all other cases. After fluid-air exchange with sterilized air was complete, intraocular gas tamponade was administered using sulfur hexafluoride (SF_6_) or perfluoropropane (C_3_F_8_), depending to the surgeon’s judgment. In patients with significant cataracts, phacoemulsification was performed before vitrectomy, with intraocular lens implantation deferred until just before fluid-air exchange. Postoperatively, patients were required to maintain face-down with prone position for at least one week.

### 2.4. Statistical Analyses

Statistical analyses were performed using IBM SPSS Statistics, version 29.0 (IBM Corp., Armonk, NY, USA). Pre- and postoperative BCVA and CFT were compared using the Wilcoxon signed-rank test. Correlations between postoperative BCVA or CFT and potential prognostic factors were first explored using Spearman correlation for continuous variables and the Mann–Whitney *U* test for dichotomous variables. These exploratory analyses were conducted to identify preliminary correlations and to screen predictors for subsequent regression analysis.

Due to the retrospective design, the number of eyes differed across baseline subgroups. To address this, correlation and regression analyses were employed using all available data, rather than artificially matching subgroup sizes. Linear regression analyses were subsequently performed on variables showing a significance level of *p* < 0.05 to determine factors correlated with postoperative outcomes. Univariable regression was first applied, followed by age-adjusted analysis to control for potential confounders. Interaction terms between age and key structural variables (FD and ellipsoid zone [EZ] disruption) were examined to assess effect modification. No significant interaction was observed (all *p* > 0.1; Table A1), indicating that the effects of FD and EZ disruption did not vary by age. However, because age distribution was markedly uneven across subgroups, age was considered a potential confounder. Therefore, univariable linear regression without age adjustment was performed as the main analysis, and age-adjusted results were presented for comparison.

To evaluate the potential influence of cataract surgery on visual and anatomical outcomes, subgroup analyses were performed comparing patients who underwent combined phaco-vitrectomy versus vitrectomy alone. Baseline characteristics were compared between the phaco-vitrectomy group and the vitrectomy-only group using the Mann–Whitney *U* test for continuous variables and Fisher’s exact test for categorical variables. Within-group preoperative and postoperative changes in BCVA and retinal thickness were assessed using the Wilcoxon signed-rank test. In addition, between-group differences in the magnitude of postoperative changes in BCVA and retinal thickness were compared using the Mann–Whitney *U* test.

## 3. Results

### 3.1. Baseline Characteristics

We retrospectively analyzed 33 eyes from 28 patients. Baseline characteristics of patients are presented in Table 1 (see Supplementary File S1 for detailed clinical parameters). Mean age was 62.4 ± 11.2 years, with 5 (15.2%) and 28 (84.8%) eyes from male and female patients, respectively. Mean preoperative BCVA was 0.74 ± 0.52 logMAR, and mean AXL was 28.48 ± 1.40 mm. Mean SE was calculated based on 17 patients, excluding those who had undergone refractive or cataract surgery. On preoperative OCT, mean FH was 408.7 ± 211.3 μm. Concomitant findings included ERM in 27 (81.8%), VMT in 17 (51.5%), FD in 7 (21.2%), and EZ disruption in 8 (24.2%) eyes. According to the ATN classification, which evaluates myopic atrophy maculopathy (MAM), MTM, and myopic neovascular maculopathy (MNM), more than one-half of the eyes were classified as A1–2, T1–2, and N0. Combined phacoemulsification was required in 23 (69.7%) eyes due to significant cataract.

### 3.2. Visual and Anatomical Outcomes

At 12 months after vitrectomy with ILM peeling and gas tamponade, mean BCVA significantly improved from 0.74 ± 0.52 to 0.30 ± 0.30 logMAR (*p* < 0.001). Mean foveal thickness, measured preoperatively as FH including schitic cavities (408.7 ± 211.3 µm), significantly decreased to a postoperative CFT of 211.2 ± 58.5 µm (*p* < 0.001) (Table 2). One eye developed FTMH 6 months after primary surgery. Because MH was not closed at 12 months, postoperative BCVA and CFT could not be assessed. Hence, this case was excluded from the analysis of surgical outcomes at 12 months. Overall surgical success rate was 97.0%, indicating highly favorable surgical results.

**Figure 2:**
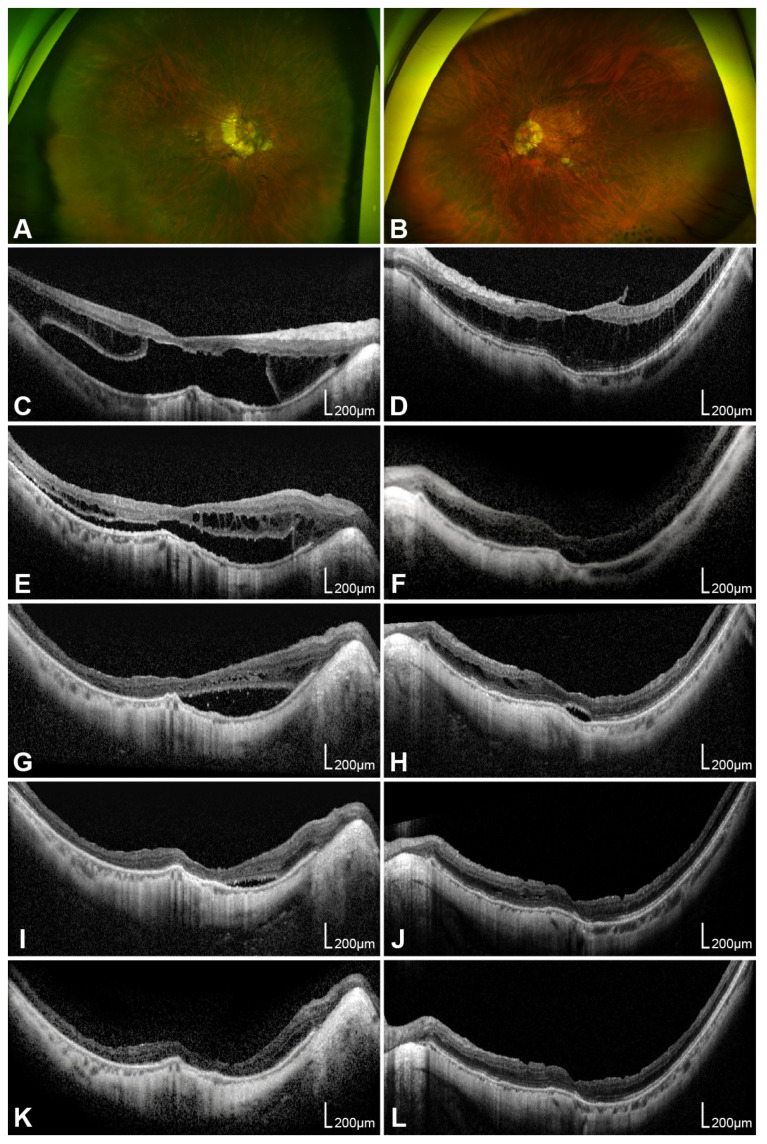
A representative case of myopic traction maculopathy involving both eyes in a single patient who underwent pars plana vitrectomy with internal limiting membrane peeling and gas tamponade. Preoperative wide-field fundus photographs and optical coherence tomography (OCT) images of the right eye (**A**,**C**) and left eye (**B**,**D**) are shown. Serial OCT images of the right eye with foveal detachment are presented in chronological order: (**E**) 1, (**G**) 5, (**I**) 10, and (**K**) 16 months postoperatively. Serial OCT images of the left eye with epiretinal membrane and vitreomacular traction are arranged in the same sequence: (**F**) 1, (**H**) 3, (**J**) 8, and (**L**) 15 months postoperatively.

Representative cases of MTM in both eyes of a single patient after PPV with ILM peeling and gas tamponade are shown in Figure 2. In the right eye with FD, gradual retinal reattachment and stabilization of foveal thickness were observed for up to 16 months postoperatively, accompanied by functional BCVA improvement. In the fellow eye with ERM- and VMT-associated tractional macular schisis, observations were initially made. However, schisis progression and visual deterioration occurred after 21 months, leading to the decision for surgical intervention. OCT follow-up demonstrated gradual anatomical resolution with corresponding visual improvement during 15 months of postoperative follow-up.

### 3.3. Prognostic Factors

We assessed potential prognostic factors for functional (postoperative BCVA) and anatomical (final CFT) outcomes (Table 3). Spearman correlation analysis revealed that worse postoperative BCVA significantly correlated with older age (*p* = 0.020), worse preoperative BCVA (*p* = 0.002), and more advanced structural deformation, including higher FH (*p* = 0.032), higher MAM (*p* = 0.033), MTM (*p* = 0.020), and ATN total grade (*p* = 0.030). Thinner postoperative CFT correlated with older age (*p* = 0.040), longer AXL (*p* = 0.004), higher MAM (*p* = 0.002), MTM (*p* = 0.038), and ATN total grade (*p* = 0.015). Mann–Whitney *U* test analysis revealed that postoperative BCVA was significantly worse in the eyes with FD (*p* = 0.030) or EZ disruption (*p* = 0.047). Postoperative CFT was significantly thinner in eyes with FD (*p* = 0.036), with EZ disruption (*p* = 0.045), and from female patients (*p* = 0.011).

Overall, older age, higher MAM, MTM, ATN total grade, and presence of FD or EZ disruption influenced both poorer postoperative BCVA and thinner CFT. Notably, worse preoperative BCVA and greater FH correlated with poorer postoperative visual outcomes, whereas female sex and longer AXL correlated with thinner postoperative CFT.

To identify predictive factors for postoperative outcomes, univariable linear regression was performed using variables that were significant in previous analyses (Table 4). Better postoperative BCVA was significantly correlated with better preoperative BCVA (*p* < 0.001) and lower FH (*p* < 0.001). The higher MTM grade (*p* = 0.022), the presence of FD (*p* = 0.024), and EZ disruption (*p* = 0.043) were associated with poorer postoperative visual outcomes. Thinner postoperative CFT was significantly associated with longer AXL (*p* = 0.004), higher preoperative MAM grade (*p* = 0.003), higher MTM grade (*p* = 0.045), and higher ATN total grade (*p* = 0.015). The presence of FD (*p* = 0.029), and EZ disruption (*p* = 0.043) were also correlated with less favorable anatomical recovery.

Age-adjusted univariable linear regression analyses showed similar overall trends, although statistical significance was attenuated in some comparisons (Table 5). In the age-adjusted univariable linear regression model, worse postoperative BCVA significantly correlated with worse preoperative BCVA (*B* = 0.317, *p* = 0.002) and higher FH (*B* = 0.001, *p* < 0.001), whereas thinner postoperative CFT significantly correlated with longer AXL (*B* = −17.366, *p* = 0.021) and higher MAM grade (*B* = −31.943, *p* = 0.019) (Table 5). Collectively, poorer preoperative BCVA and greater FH, reflecting more severe schisis or traction, correlated with unfavorable functional outcomes, whereas longer AXL and advanced MAM grade, indicative of central atrophy, predicted thinner postoperative CFT.

### 3.4. Influence of Combined Cataract Surgery on Outcomes

Baseline characteristics were comparable between the phaco-vitrectomy and vitrectomy-only groups (all *p* > 0.05) (Table 6).

Within-group BCVA improvement reached statistical significance in the phaco-vitrectomy group (*p* < 0.001), whereas no statistically significant improvement was observed in the vitrectomy-only group (*p* = 0.092). Both groups showed significant improvement in postoperative retinal thickness (both *p* < 0.001). When comparing the magnitude of postoperative changes between groups, the BCVA gain was significantly greater in the phaco-vitrectomy group than in the vitrectomy-only group (*p* = 0.017), whereas the degree of retinal thickness change did not differ significantly between groups (*p* = 0.615) (Table 7).

## 4. Discussion

To our knowledge, this study is the first to systematically analyze preoperative OCT parameters, aiming to evaluate long-term functional and anatomical outcomes as well as assess prognostic factors for vitrectomy in MTM without FTMH. In our cohort, patients with better preoperative BCVA and lower preoperative FH demonstrated superior postoperative BCVA, whereas longer AXL, advanced MAM and ATN total grade correlated with thinner postoperative CFT. Presence of FD, EZ disruption, and higher MTM grade correlated with both poorer postoperative BCVA and reduced postoperative CFT. By contrast, the presence of ERM and VMT did not significantly influence either functional or anatomical outcomes. Overall, mean BCVA improved and mean foveal thickness decreased significantly 12 months postoperatively.

Linear regression analysis identified preoperative BCVA as a positive and FH as a negative prognostic factor for functional outcomes, whereas AXL, MAM grade, and ATN total grade were identified as negative predictors of anatomical outcomes. In addition, the presence of FD, EZ disruption, and MTM grade appeared to be negative predictive factors for both postoperative functional and structural outcomes. In the age-adjusted linear regression analysis, poorer preoperative BCVA and greater FH, reflecting more severe schisis or traction, correlated with unfavorable functional outcomes, whereas longer AXL and advanced MAM grade, indicative of central atrophy, predicted thinner postoperative CFT.

Nevertheless, visual acuity outcomes should be interpreted with caution, as they may have been influenced by lens-related factors, including combined cataract surgery, post-vitrectomy cataract progression, and posterior capsule opacity (PCO). These factors may limit the extent to which postoperative BCVA reflects the effect of vitrectomy alone.

### 4.1. Importance and Safety of ILM Peeling

Current debates in the management of MTM mainly concern the surgical technique and optimal timing of intervention. Among these, PPV remains the most commonly performed procedure, because it effectively relieves vitreoretinal traction responsible for FD. Key issues include total versus fovea-sparing ILM peeling, because secondary FTMH has been reported after total ILM peeling (Ho et al. [22], 28.6% and Shimada et al. [23], 16.7%).

Proper stiffness of the ILM is essential for maintaining mechanical balance at the vitreoretinal interface. However ultrastructural studies have demonstrated increased ILM rigidity in highly myopic eyes with MTM, attributable to reactive Müller cell gliosis and increased fibrous astrocyte content [26]. This abnormal stiffness generates centripetal traction on the retina, promoting retinal splitting and tractional changes such as MTM. Moreover, contraction of the remnant preretinal vitreous cortex also contributes to the development of foveoschisis and FD in eyes with posterior staphyloma [24,27].

Based on these considerations, complete ILM peeling was performed in most cases to achieve adequate traction release in this study. A fovea-sparing approach was selectively applied in eyes with highly severe traction as extreme foveal thinning or outer lamellar holes. Despite concerns regarding postoperative FTMH formation, only one case developed FTMH, yielding a surgical success rate of 97.0%, which is comparable to or higher than those reported in previous studies (from 82.5% [28] to 89.5% [29]).

### 4.2. Prognostic Factors for Functional and Anatomical Outcomes

Preoperative BCVA showed a strong positive correlation with postoperative BCVA and remained a consistent predictor of postoperative outcomes in regression analysis, consistent with prior studies that identified baseline visual acuity as the strongest predictor of postoperative visual outcomes [12,13,30,31]. AXL negatively correlated with postoperative CFT but not BCVA, consistent with some reports [13,29] but in contrast to others linking longer AXL with poorer visual outcomes [28,31].

In highly myopic eyes, longer AXL reflects greater posterior scleral elongation and is associated with more severe myopic maculopathy [32] and macular atrophy [5]. Accordingly, a higher MAM grade was also negatively correlated with postoperative CFT in our study. Progressive atrophic changes in eyes with longer AXL and higher MAM grade are characterized by photoreceptor loss and retinal thinning, which may limit postoperative anatomical restoration [5] providing a plausible explanation for the observed association between greater myopic severity and thinner postoperative CFT.

Overall, our findings indicate that increasing AXL and MAM grade are associated with progressively poorer anatomical outcomes, whereas baseline visual function primarily determines postoperative BCVA. This dissociation between functional and anatomical predictors persisted after age adjustment, suggesting that longer follow-up may be required to assess the long-term visual implications of postoperative anatomical thinning in eyes with advanced myopic degeneration.

In addition, the ATN total grade, reflecting the overall severity of myopic maculopathy, correlated with both functional and anatomical outcomes. However, its prognostic relevance was limited to anatomical outcomes in linear regression and was attenuated after age adjustment, suggesting an indirect, age-related effect rather than an independent predictor of postoperative recovery.

Although ERM and VMT exert macular traction, the presence of ERM and VMT did not significantly correlate with postoperative BCVA or CFT in our cohort. In contrast, MTM grade was negatively associated with both functional and anatomical outcomes in correlation analyses and univariable linear regression, indicating that increasing traction severity was related to worse postoperative BCVA and thinner postoperative CFT. However, these associations were attenuated after age adjustment, suggesting that the prognostic impact of MTM grade may be partly influenced by age-related disease progression rather than traction severity alone.

Feng et al. [13] suggests VMT as an adverse prognostic factor for visual outcome, and some studies have suggested that ERM, VMT, or partial posterior vitreous detachment (PVD) accelerates MTM progression [33,34], thereby increasing the benefit of surgery. However, definitive evidence linking preoperative VMT or ERM to visual outcomes is lacking [5,29], consistent with our study, indicating that the mere presence of ERM or VMT does not independently predict postoperative outcomes. As traction increases with advancing MTM grade, photoreceptor and Müller cell damage becomes more severe, leading to worse functional and anatomical outcomes. Even in eyes with complete PVD where neither ERM nor posterior hyaloid membrane is visible on preoperative OCT, remnants of the posterior vitreous cortex may persist, as evidenced by the intraoperative detection of premacular membranes [24,35]. Remnant vitreous and ERM contribute to retinal rigidity and perpendicular traction on the retina, which plays significant roles in the pathogenesis of MTM [5]. Consequently, we performed meticulous removal of ERM and ILM to eliminate residual traction, which likely explains the lack of impact of ERM or VMT on surgical outcomes in our cohort.

In this study, preoperative FH and postoperative CFT were intentionally evaluated as distinct parameters. FH reflects the severity of traction-induced foveal deformation at baseline, whereas CFT represents postoperative anatomical restoration of the retina. Interestingly, greater preoperative FH correlated with worse postoperative visual outcomes, and remained a significant prognostic factor in both univariable linear regression and age-adjusted analyses, underscoring its value as a negative functional prognostic marker. This contrasts with studies reporting where baseline foveal thickness did not significantly influence visual outcomes at 3, 6, or 12 months following surgery for idiopathic ERM [36,37] or myopic foveoschisis [38]. However, thicker preoperative FH reflects more advanced foveoschisis, which likely compromises photoreceptor oxygenation and nutrient delivery from the choriocapillaris, resulting in functional impairment. Therefore, earlier surgical intervention before a marked increase in FH may lead to better visual outcomes.

In our study, seven of eight patients with EZ disruption had FD, resulting in similar trends in those subgroup analyses. Groups with EZ disruption and FD demonstrated significantly worse final BCVA and thinner postoperative CFT in both correlation analyses and univariable linear regression, indicating advanced structural injuries on fovea.

Regarding EZ disruption, Fujimoto et al. [38] reported that final BCVA correlated with postoperative photoreceptor integrity rather than preoperative status. Nevertheless, numerous studies have identified preoperative photoreceptor disruption as a strong adverse prognostic factor for postoperative BCVA following vitrectomy for ERM [36,37,39,40] and MTM [29,33]. According to Inoue et al. [39], in a prospective cohort, preoperative EZ disruption reflects irreversible photoreceptor injury caused by sustained mechanical separation or shearing of photoreceptor cells, whereas transient postoperative disruption may represent reversible inflammatory changes due to breakdown of the blood–retinal barrier. In our study, six of eight eyes with EZ disruption showed partial restoration of EZ contour, whereas two remained disrupted. Even when EZ recovered anatomically, functional impairment of photoreceptors likely persisted [33], consistent with the hypothesis of Inoue et al. [39], which may explain the association with poorer postoperative BCVA and thinner postoperative CFT. And, in case of FD, the photoreceptor layer is physically separated from the underlying tissue and sustains more extensive damage beyond EZ disruption, which may explain why FD, like EZ disruption, serves as a poor prognostic factor for both functional and anatomical outcomes.

Although the associations of FD and EZ disruption with postoperative outcomes lost statistical significance after age adjustment, these remain clinically meaningful, as both features indicate advanced foveal structural damage that limits postoperative visual and anatomical recovery. The attenuation of statistical significance should be interpreted in the context of marked subgroup imbalance and limited statistical power, rather than absence of biological relevance, as eyes with FD or EZ disruption were substantially older and few in number. Similarly, while age and sex were negatively associated with postoperative outcomes in correlation analysis, neither remained significant in regression models, suggesting that these demographic factors reflect underlying disease severity rather than independent prognostic determinants.

Ikuno et al. [12] and Kumagai et al. [31] reported that eyes with FD resulted in greater BCVA gains compared to eyes without FD, and proposed that FD represents the most appropriate surgical indication because patients with FD may derive maximal benefit from timely intervention. This is reasonable, given that reattachment of foveal photoreceptors is directly linked to visual recovery. However, the presence of FD was a well-established predictor of poor postoperative BCVA [13,28,30], reflecting advanced foveal traction and irreversible outer photoreceptor damage. Despite the small number of eyes with FD in our cohort and uneven age distribution, our data support presence of FD and EZ disruption as key markers of advanced MTM severity, serving as potential—but not independent—prognostic factors for postoperative functional and anatomical outcomes and underscore the importance of considering surgical intervention before irreversible foveal damage occurs.

### 4.3. Implications of Combined Cataract Surgery for Visual Outcomes

Since cataract progression and cataract surgery can substantially influence postoperative visual acuity, the visual acuity outcomes in this study should be interpreted cautiously and conservatively. We acknowledge that postoperative visual acuity cannot be interpreted as reflecting the effect of vitrectomy alone, as there are two potential confounding variables: cataract surgery performed at the time of vitrectomy and cataract progression following vitrectomy in eyes without combined cataract surgery (Supplementary File S2).

To address this concern, we evaluated the impact of combined phacoemulsification on surgical outcomes. Baseline characteristics, including preoperative OCT parameters and ATN grades, were comparable between the phaco-vitrectomy and vitrectomy-only groups, suggesting that the two groups were well balanced. In the phaco-vitrectomy group, a statistically significant improvement in BCVA was observed, suggesting that concomitant cataract surgery likely contributed to the visual improvement. Furthermore, the magnitude of BCVA gain was significantly greater in the phaco-vitrectomy group than in the vitrectomy-only group. This finding suggests that combined cataract surgery may have contributed, at least in part, to the overall visual improvement observed in this study, and the visual acuity results should therefore be interpreted with caution.

Nevertheless, both groups demonstrated significant anatomical improvement postoperatively, and the degree of retinal thickness reduction was comparable between groups, although further studies are needed to clarify the independent effect of vitrectomy on visual outcomes.

Moreover, PCO following cataract surgery may have influenced postoperative visual acuity in the combined phaco-vitrectomy group. In our cohort, PCO developed in eight eyes, and two of these underwent Nd:YAG laser capsulotomy within one year postoperatively, with no notable change in BCVA after the procedure. The clinical data for these patients are provided in Supplementary File S3. Although PCO did not appear to have a substantial impact on visual outcomes in our cohort, its potential confounding effect should be considered when interpreting the BCVA results.

### 4.4. Optimal Timing for Surgery

MTM progresses slowly but can eventually lead to irreversible visual loss. Therefore, serial OCT monitoring is essential, since early progression is hardly detected on fundus examination. As demonstrated above, timely surgical approach plays a critical role in preserving long-term visual function and quality of life in patients.

The key clinical challenge, therefore, is determining the optimal timing of surgery in eyes with MTM prior to FD onset to achieve the most favorable functional and anatomical results. Clinically, surgery has been considered in highly myopic eyes when schisis rapidly worsens with subjective visual symptoms, when VMT or ERM is present, or once FD has occurred [5]. However, our results suggest that waiting until FD develops may compromise optimal outcomes. We found that better preoperative visual acuity leads to superior postoperative outcomes, while the presence of FD, along with progression of macular traction and atrophy, correlated with unfavorable anatomical and functional recovery.

EZ disruption, presence of FD, and increasing tractional grade are all structural changes that occur during MTM progression. Increased preoperative traction correlated with greater preoperative FH, which in turn predicted worse postoperative visual acuity. Based on these findings, surgical intervention may be justified even in eyes without FD if patients complain of progressive visual decline, if schisis worsens with increasing traction (i.e., progressive FH increase), or prior to the development of EZ disruption. To optimize postoperative visual acuity and foveal anatomy, surgery should be considered according to these criteria, and earlier intervention may be particularly beneficial in eyes with longer AXL, macular atrophy, and older age.

Notably, one patient developed secondary FTMH despite the absence of preoperative FD, EZ disruption, ERM, or VMT and with relatively preserved ATN grade (A2T2N0) and FH (284 µm). A macular hole appeared 6 months postoperatively, and despite additional surgeries, including autologous platelet injection, anatomical closure was not achieved. This case illustrates that secondary FTMH can occur even in eyes without conventional risk factors, highlighting the unpredictable nature of MTM progression and the challenges in determining optimal surgical timing.

### 4.5. Limitations and Further Research Directions

Several limitations should be acknowledged. First, the retrospective design and small sample size resulted in uneven distribution across subgroups, including those with versus without FD, EZ disruption, and ERM. This imbalance reflects the heterogeneous clinical spectrum of MTM, as FD and EZ disruption occur in more advanced stages, and the inherent limitations of retrospective study design. Consequently, several variables that appeared to be significant prognostic factors in the univariable regression analysis were no longer statistically significant after adjusting for age.

Second, postoperative visual acuity may have been influenced by lens-related factors, including cataract surgery performed at the time of vitrectomy and potential cataract progression following vitrectomy. The greater BCVA gain observed in the phaco-vitrectomy group suggests that combined cataract surgery may have contributed to the overall visual improvement. Therefore, the visual acuity outcomes in this study should be interpreted cautiously and conservatively, and the independent effect of vitrectomy on visual recovery warrants further investigation. In addition, PCO following cataract surgery may have influenced postoperative BCVA in the phaco-vitrectomy group, as discussed in Section 4.3. Its potential confounding effect warrants consideration.

Third, as this was a single-center study and all surgeries were performed by a single surgeon, the generalizability of our findings may be limited across different clinical settings. Third, although surgical procedures were standardized, minor variations in surgical technique (such as the extent of ILM peeling, type and concentration of gas tamponade, and intraoperative decision-making) may have introduced potential confounding. Therefore, long-term prospective multicenter studies with larger, age-balanced cohorts and standardized surgical protocols are warranted to validate these findings and to further clarify prognostic factors and the optimal timing of surgical intervention in MTM.

## 5. Conclusions

In eyes with MTM without FTMH, preoperative visual acuity, AXL, and OCT parameters are closely associated with postoperative functional and anatomical outcomes. Better preoperative visual acuity and lower FH predict superior postoperative BCVA, whereas longer AXL and higher MAM and ATN total grades are associated with thinner postoperative CFT. The presence of FD, EZ disruption, and advanced MTM grade indicate more severe disease and correlate with poorer outcomes, underscoring the importance of considering surgical intervention before FD develops. These findings support early surgical consideration in eyes with progressive macular traction, EZ disruption, evolving macular atrophy, or visual decline (even in the absence of FD) and highlight the importance of serial OCT monitoring and individualized surgical timing based on preoperative structural and functional assessments.

## Figures and Tables

**Table 1:** Characteristics of study population (*n* = 33 eyes).

Baseline Parameters	Values
Age (years)	62.4 ± 11.2 (39–75)
Sex, male:female, *n* (%)	5:28 (15.2%:84.8%)
BCVA (logMAR)	0.74 ± 0.52
SE (diopter)	−12.54 ± 5.28 (*n* = 17)
Refractive Surgery history, *n* (%)	9 (27.3%)
Axial length (mm)	28.48 ± 1.40
Lens status, *n* (%)	
Cataract	25 (75.8%)
No cataract	1 (3.0%)
Pseudophakia	7 (21.2%)
**Preoperative OCT parameters**	
Foveal height(μm)	408.7 ± 211.3
ERM, *n* (%)	27 (81.8%)
VMT, *n* (%)	17 (51.5%)
Foveal detachment, *n* (%)	7 (21.2%)
EZ disruption, *n* (%)	8 (24.2%)
ATN classification, *n* (%)	
MAM grade 0:1:2:3:4	1:21:6:5:0 (3.0%:63.6%:18.2%:15.2%:0%)
MTM grade 0:1:2:3:4:5	0:16:10:7:0:0 (0%:48.5%:30.3%:21.2%:0%:0%)
MNM grade 0:1:2a:2s	30:1:0:2 (90.9%:3.0%:0%:6.1%)
**Operational parameters (%)**	
Combined Phacoemulsification, *n* (%)	23 (69.7%)
Gas, SF_6_: C_3_F_8_, *n* (%)	2:31 (6.1%:93.9%)

Values are presented as mean ± standard deviation or number (%). BCVA = best-corrected visual acuity; logMAR = logarithm of the minimum angle of resolution; SE = spherical equivalent; OCT = optical coherence tomography; ERM = epiretinal membrane; VMT = vitreomacular traction; EZ = ellipsoid zone; MAM = myopic atrophy maculopathy; MTM = myopic traction maculopathy; MNM = myopic neovascular maculopathy; SF_6_ = sulfur hexafluoride; C_3_F_8_ = perfluoropropane.

**Table 2:** Comparison of preoperative and postoperative visual and anatomical parameters (*n* = 32).

	Preoperative	Postoperative (12 Months)	Change	*p* Value
BCVA (logMAR)	0.744 ± 0.52	0.299 ± 0.30	−0.445 ± 0.41	<0.001
Foveal thickness (μm)	408.7 ± 211.3 (FH)	211.2 ± 58.5 (CFT)	−201.4 ± 216.0	<0.001

Values are presented as mean ± standard deviation. One eye that developed a full-thickness macular hole (FTMH) 6 months postoperatively was excluded from the 12-month postoperative analysis (final *n* = 32). BCVA = best-corrected visual acuity; FH = foveal height; CFT = central foveal thickness; *p* value from the Wilcoxon signed-rank test between preoperative and postoperative BCVA or CFT.

**Table 3:** Factors related with functional and anatomical outcomes.

		Postop BCVA (logMAR)		Postop CFT (μm)	
		*r*	*p* value *^a^*	*r*	*p* value *^a^*
Age (years)		0.409	0.020 *	−0.365	0.040 *
Preop BCVA (logMAR)		0.528	0.002 *	−0.276	0.126
SE (diopter)		−0.187	0.304	0.194	0.286
Axial length (mm)		0.283	0.117	−0.494	0.004 *
Foveal height (μm)		0.380	0.032 *	−0.060	0.743
MAM grade		0.378	0.033 *	−0.528	0.002 *
MTM grade		0.409	0.020 *	−0.368	0.038 *
MNM grade		0.081	0.659	−0.200	0.272
ATN total grade		0.385	0.030 *	−0.425	0.015 *
		Mean ± SD	*p* value *^b^*	Mean ± SD	*p* value *^b^*
Gender	M (*n* = 5)	0.564 ± 0.433		277.6 ± 53.0	
	F (*n* = 27)	0.254 ± 0.257	0.078	198.9 ± 52.7	0.011 *
Lens status	Cataract (*n* = 25)	0.287 ± 0.325		215.1 ± 56.7	
	No cataract or Pseudophakia (*n* = 7)	0.358 ± 0.230	0.310	197.3 ± 71.6	0.274
Phacoemulsification	Yes (*n* = 23)	0.281 ± 0.336		214.7 ± 59.2	
	No (*n* = 9)	0.357 ± 0.211	0.218	202.3 ± 62.8	0.379
ERM	Present (*n* = 27)	0.297 ± 0.315		215.0 ± 61.2	
	Absent (*n* = 5)	0.330 ± 0.274	0.693	191.0 ± 49.5	0.392
VMT	Present (*n* = 17)	0.381 ± 0.339		212.9 ± 59.5	
	Absent (*n* = 15)	0.213 ± 0.242	0.112	209.3 ± 61.4	0.880
Foveal detachment	Present (*n* = 7)	0.528 ± 0.373		168.6 ± 68.5	
	Absent (*n* = 25)	0.239 ± 0.257	0.030 *	223.2 ± 52.1	0.036 *
EZ disruption	Present (*n* = 8)	0.489 ± 0.362		174.8 ± 65.8	
	Absent (*n* = 24)	0.240 ± 0.263	0.047 *	223.4 ± 53.2	0.045 *
Gas tamponade	SF_6_ (*n* = 2)	0.049 ± 0.069		218.5 ± 2.1	
	C_3_F_8_ (*n* = 30)	0.319 ± 0.307	0.180	210.7 ± 61.4	1.000

Values are presented as mean ± standard deviation (SD). Statistically significant differences (*p* < 0.05) are indicated in bold and marked with an asterisk (*). *^a^* Spearman correlation analysis; *^b^* Mann–Whitney *U* test. BCVA = best-corrected visual acuity; logMAR = logarithm of the minimum angle of resolution; SE = spherical equivalent; CFT = central foveal thickness; ERM = epiretinal membrane; VMT = vitreomacular traction; EZ = ellipsoid zone; MAM = myopic atrophy maculopathy; MTM = myopic traction maculopathy; MNM = myopic neovascular maculopathy; SF_6_ = sulfur hexafluoride; C_3_F_8_ = perfluoropropane.

**Table 4:** Preoperative factors correlated with functional and anatomical outcomes after vitrectomy: univariable linear regression.

	Postoperative BCVA (logMAR)			Postoperative CFT (μm)		
	*B*	*p* Value	95% CI	*B*	*p* Value	95% CI
Preop BCVA (logMAR)	0.356	<0.001 *	0.189 to 0.524	−12.588	0.540	−54.036 to 28.860
Axial length (mm)	0.058	0.137	−0.019 to 0.135	−20.616	0.004 *	−34.193 to −7.038
Foveal height (μm)	0.001	<0.001 *	<0.001 to 0.001	0.025	0.616	−0.077 to 0.127
MAM grade	0.102	0.138	−0.035 to 0.239	−37.588	0.003 *	−61.453 to −13.723
MTM grade	0.152	0.022 *	0.024 to 0.280	−26.139	0.045 *	−51.610 to −0.668
ATN total grade	0.057	0.063	−0.003 to 0.118	−14.392	0.015 *	−25.721 to −3.062
Foveal detachment	0.289	0.024 *	0.041 to 0.537	−54.589	0.029 *	−103.266 to −5.912
EZ disruption	0.249	0.043 *	0.008 to 0.490	−48.625	0.043 *	−95.620 to −1.630

Statistically significant differences (*p* < 0.05) are indicated in bold and marked with an asterisk (*). *p*-values were obtained using the linear regression model. *B* = regression coefficient; BCVA = best-corrected visual acuity; CFT = central foveal thickness; CI = confidence interval; logMAR = logarithm of the minimum angle of resolution; MAM = myopic atrophy maculopathy; MTM = myopic traction maculopathy; EZ = ellipsoid zone.

**Table 5:** Preoperative factors correlated with functional and anatomical outcomes after vitrectomy: age-adjusted linear regression.

	Postoperative BCVA (logMAR)			Postoperative CFT (μm)		
	*B*	*p* Value	95% CI	*B*	*p* Value	95% CI
Preop BCVA (logMAR)	0.317	0.002 *	0.132 to 0.502	5.694	0.792	−38.033 to 49.421
Axial length (mm)	0.029	0.458	−0.051 to 0.110	−17.366	0.021 *	−31.974 to −2.757
Foveal height (μm)	0.001	<0.001 *	<0.001 to 0.001	0.062	0.213	−0.037 to 0.161
MAM grade	0.046	0.525	−0.100 to 0.192	−31.943	0.019 *	−58.352 to −5.533
MTM grade	0.109	0.118	−0.029 to 0.246	−18.278	0.187	−45.913 to 9.356
ATN total grade	0.033	0.310	−0.033 to 0.099	−11.181	0.081	−23.815 to 1.452
Foveal detachment	0.202	0.137	−0.068 to 0.472	−39.865	0.139	−93.389 to 13.659
EZ disruption	0.186	0.126	−0.055 to 0.428	−37.830	0.116	−85.577 to 9.918

Statistically significant differences (*p* < 0.05) are indicated in bold and marked with an asterisk (*). *p*-values were obtained using the linear regression model. BCVA = best-corrected visual acuity; CFT = central foveal thickness; logMAR = logarithm of the minimum angle of resolution; MAM = myopic atrophy maculopathy; MTM = myopic traction maculopathy; EZ = ellipsoid zone.

**Table 6:** Subgroup analysis for baseline characteristics between the phaco-vitrectomy and vitrectomy-only group.

	Phaco-Vitrectomy Group	Vitrectomy-Only Group	*p* Value
Age	63.0 ± 9.6	58.3 ± 10.1	0.200 ^a^
Preop BCVA (logMAR)	0.843 ± 0.575	0.531 ± 0.326	0.175 ^a^
Axial length (mm)	28.39 ± 1.31	28.54 ± 1.76	0.900 ^a^
Preop Foveal height (μm)	409.1 ± 229.0	421.6 ± 194.3	0.900 ^a^
ERM, *n* (present:absent)	20:3	7:2	0.604 ^b^
VMT, *n* (present:absent)	12:11	5:4	1 ^b^
FD, *n* (present:absent)	4:19	3:6	0.370 ^b^
EZ disruption, *n* (present:absent)	4:19	4:5	0.176 ^b^
MAM grade	1.39 ± 0.78	1.56 ± 0.88	0.691 ^a^
MTM grade	1.74 ± 0.75	1.67 ± 1.0	0.648 ^a^
MNM grade	0.13 ± 0.46	0.22 ± 0.67	0.836 ^a^
ATN total grade	3.26 ± 1.63	3.44 ± 2.19	0.807 ^a^

Values are presented as mean ± standard deviation or number. ^a^ Mann–Whitney *U* test, ^b^ Fisher exact test. BCVA = best-corrected visual acuity; logMAR = logarithm of the minimum angle of resolution; ERM = epiretinal membrane; VMT = vitreomacular traction; FD = foveal detachment; EZ = ellipsoid zone; MAM = myopic atrophy maculopathy; MTM = myopic traction maculopathy; MNM = myopic neovascular maculopathy; CFT = central foveal thickness.

**Table 7:** Comparison of BCVA gain and retinal thickness change between phaco-vitrectomy group and vitrectomy-only group.

	BCVA (logMAR)				Retinal Thickness (μm)			
	Preop BCVA	Postop BCVA	BCVA Change	*p ^a^*	Preop FH (μm)	Postop CFT (μm)	Thickness Change	*p ^a^*
Phaco-vitrectomy	0.84 ± 0.57	0.28 ± 0.34	−0.56 ± 0.42	**<0.001**	409.1 ± 229.0	214.7 ± 59.2	−194.4 ± 228.6	**<0.001**
Vitrectomy-only	0.53 ± 0.33	0.36 ± 0.21	−0.17 ± 0.25	0.092	421.6 ± 194.3	202.3 ± 62.8	−219.2 ± 205.8	**0.019**
*p* value *^b^*			**0.017**				0.615	

Statistically significant differences (*p* < 0.05) are indicated in bold. *^a^* Wilcoxon signed-rank test, *^b^* Mann–Whitney *U* test. BCVA = best-corrected visual acuity; FH = foveal height; CFT = central foveal thickness; logMAR = logarithm of the minimum angle of resolution.

## Data Availability

The complete dataset presented in this study is not publicly available due to privacy and ethical restrictions. However, data may be available from the corresponding author upon reasonable request. Anonymized key clinical parameters for all study participants are available in Supplementary Materials (Supplementary File S1). Visual outcomes and cataract surgery history for phakic eyes in the vitrectomy-only group are provided in Supplementary File S2. Clinical data regarding posterior capsule opacity and Nd:YAG laser capsulotomy are provided in Supplementary File S3.

## Supplementary Materials

The following supporting information can be downloaded at: https://www.mdpi.com/article/10.3390/life16020356/s1.

## Abbreviations

The following abbreviations are used in this manuscript:

SE	Spherical equivalent
D	Diopters
AXL	Axial length
PM	Pathologic myopia
MTM	Myopic traction maculopathy
RD	Retinal detachment
FD	Foveal detachment
LMH	Lamellar macular hole
FTMH	Full-thickness macular hole
MHRD	Macular hole-related retinal detachment
PPV	Pars plana vitrectomy
ILM	Internal limiting membrane
OCT	Optical coherence tomography
VMT	Vitreomacular traction
ERM	Epiretinal membrane
BCVA	Best-corrected visual acuity
logMAR	Logarithm of the minimum angle of resolution
FH	Foveal height
RPE	Retinal pigment epithelium
CFT	Central foveal thickness
SF_6_	Sulfur hexafluoride
C_3_F_8_	Perfluoropropane
EZ	Ellipsoid zone
MAM	Myopic atrophy maculopathy
MNM	Myopic neovascular maculopathy
PVD	Posterior vitreous detachment
CI	Confidence interval

## Appendix A

**Table A1:** Preoperative factors associated with functional and anatomical outcomes after vitrectomy: linear regression with age-adjusted and interaction terms.

	Postoperative BCVA (logMAR)				Postoperative CFT (μm)			
	*B*	*p ^a^*	95% CI	*p ^b^*	*B*	*p ^a^*	95% CI	*p ^b^*
Preoperative BCVA	0.291	**0.005 ***	0.097 to 0.485	0.345	5.232	0.820	−41.330 to 51.794	0.945
Axial length	0.034	0.420	−0.051 to 0.120	0.717	−19.397	**0.016 ***	−34.867 to −3.928	0.396
Foveal height	0.001	**0.002 ***	<0.001 to 0.001	0.660	0.065	0.211	−0.039 to 0.169	0.795
MAM grade	0.046	0.568	−0.116 to 0.208	0.998	−25.955	0.076	−54.798 to 2.888	0.300
MTM grade	0.101	0.195	−0.055 to 0.258	0.830	−18.415	0.239	−49.790 to 12.961	0.984
ATN total grade	0.03	0.456	−0.051 to 0.110	0.869	−11.530	0.137	−26.947 to 3.887	0.934
Foveal detachment	0.430	0.063	−0.025 to 0.886	0.215	−11.629	0.797	−103.537 to 80.278	0.443
EZ disruption	0.203	0.117	−0.054 to 0.461	0.660	−34.437	0.177	−85.319 to 16.446	0.655

Significant differences (*p* < 0.05) are indicated in bold and marked with an asterisk (*). *a* = *p* value for age-adjusted univariable linear regression analysis; *b* = *p* value for interaction term. *B* = regression coefficient; BCVA = best-corrected visual acuity; logMAR = logarithm of the minimum angle of resolution; CFT = central foveal thickness; CI = confidence interval; MAM = myopic atrophy maculopathy; MTM = myopic traction maculopathy; EZ = ellipsoid zone.
